# A comparative study of gender differences in healthy office building strategies

**DOI:** 10.3389/fpsyg.2023.1146260

**Published:** 2023-11-27

**Authors:** Xiaohuan Xie, Ruobing Wang, Zhonghua Gou, Shan Chen

**Affiliations:** ^1^Shenzhen Key Laboratory of Built Environment Optimization, Shenzhen University, Shenzhen, China; ^2^School of Architecture and Urban Planning, Shenzhen University, Shenzhen, China; ^3^School of Urban Design, Wuhan University, Wuhan, China

**Keywords:** healthy office buildings, evaluation system, subjective action, objective execution difficulty, theory of planned behavior, gender differences

## Abstract

**Background:**

The health of office workers has become a major concern under the pressure of increasingly fierce job competition. As countries have gradually promoted healthy buildings, there is an urgent need to create and construct healthy office environments. Although the WELL Building Standard proposed management and design strategies based on the principles of health and medicine, it does not consider group characteristics or gender differences.

**Purpose:**

This study aims to apply the theory of planned behavior to healthy building design and supplement the important role of gender and group characteristics in behavioral guidance based on architectural strategies and user behaviors to improve the relevant building evaluation system.

**Methods:**

This study adopted a questionnaire survey and structural equation model. Four WELL-certified healthy office buildings in Nanshan District, Shenzhen, were selected for the survey. Based on the theory of planned behavior, structural equation models for men and women were established, compared, and analyzed. The factors affecting the health behaviors of the two groups and the actual effectiveness of various building optimization strategies were discussed, and an optimization direction for gender differences was proposed.

**Results:**

The findings indicated differences between male and female staff in their individual characteristics and implementation of health behaviors. Management strategies, subjective design strategies in assistance and guidance, and objective design strategies in spatial planning can promote the health behaviors of the two groups. However, the design strategies of result feedback and detail optimization only appeared to have a significant positive effect on female staff, whereas the intelligent automation design strategies only had an obvious intervention effect on men’s health behaviors.

**Significance:**

This study found that the theory of planned behavior in the field of social psychology could be applied to relevant research on architectural design and emphasized the influence of gender. It can not only provide the optimization direction for the evaluation standards of relevant healthy buildings but also promote the implementation of health behaviors in office groups and provide new ideas for promoting the development of healthy buildings.

## 1. Introduction

COVID-19 has caused society to realize the importance of health. However, multiple studies have shown that chronic diseases caused by health-risk behaviors such as lack of physical inactivity and sleep, poor eating habits, excessive psychological stress, and smoking pose a more urgent threat to public health worldwide than the severity of the pandemic (Lloyd-Jones et al., 2010; Soriano et al., 2019). Therefore, after recognizing the close relationship between health behaviors and individual health, it will be pivotal to hold discussions about the influencing mechanisms of health behaviors and strengthen health behavior intervention methods to improve public health (Spring et al., 2012).

In addition, according to the U.S. Environmental Protection Agency (EPA), people spend more than 90% of their time in buildings (An et al., 2016), and previous research has demonstrated that the built environment is closely linked to promoting individual health behaviors (Namgung et al., 2019; Tcymbal et al., 2020). Therefore, it is particularly important to promote the health behaviors of contemporary people in their daily lives by creating healthy and comfortable building environments. The concept of healthy buildings has emerged to achieve this goal.

The WELL Building Standard was developed by Delos in the USA. Certification and launching were performed by third-party organizations, the International WELL Building Institute (IWBI) and the Green Building Certification Institute (GBCI) [(IWBI) tIWBI, 2018]. As the first “people-oriented” building evaluation standard in the world, it not only emphasizes energy conservation and environmental protection for buildings and the effects of green-based buildings on the environment but also pays attention to the environment inside buildings and the health needs of building users. Simultaneously, from a medical perspective, a healthy and comfortable building environment for building users should be created [(IWBI) tIWBI, 2018]. There have been three main research directions based on the current literature on WELL healthy buildings. The first is the comparative study of building standards [including the self-development comparison of standards (Qiu and Chen, 2018; Yuan et al., 2019; Luo et al., 2020), the comparison of healthy building standards in various countries (Xie et al., 2018; Liu et al., 2019; Wang et al., 2020), and the comparison between green buildings and healthy building standards (Zhang et al., 2018)]. The second consists of case analysis and practical application based on healthy building evaluation standards (Jia, 2019; Zhang, 2019; Luo et al., 2020; Ning, 2020). The third discusses the benefits of healthy buildings and how to improve the hygiene and safety of the project environment (Lee et al., 2013; Sekhar et al., 2015; Hu, 2021; Zhang, 2021). In general, since the WELL standard was proposed, it has been only a short period of time, so the standard focuses on the process and project practice of promoting healthy building standards, and a small part involves its impact on health. Therefore, the research on the effectiveness of the proposed strategies on users’ health behaviors must be deepened, especially how to optimize the scoring system for existing standards to promote the implementation of multiple health behaviors. In addition, the WELL Health Standard is based on a broad range of scientific and medical theories that must be designed and promoted with a wide range of users, technologies, and policies in mind (Brambilla et al., 2020); therefore, its consideration of special groups and complex user behavior mechanisms remains to be explored.

Recently, office groups have gradually attracted public attention. In the context of fast-paced life and work, this largely sedentary group spends most of their time sitting in the office to finish their work, resulting in high work pressure, little leisure time, and generally poor health conditions (Xie et al., 2021, 2022). Currently, the literature focusing on this group has mainly discussed how to improve its satisfaction and productivity (Al Horr et al., 2016; Yeom et al., 2020) rather than promote its health behaviors. With the occurrence of public health incidents and the increasing popularity of dense workspaces and high-rise buildings, people have gradually found that poor and inadequate workplaces affect workers’ health. This discovery has led to increasing discussions on sick building syndrome (SBS) (Lee et al., 2018; Sarkhosh et al., 2021; Wang et al., 2022). Existing relevant studies have proven that the attitude and health cognition level of individual employees (Landais et al., 2022), physical environment of the office (Zhang et al., 2021), and social environment (Zhang et al., 2021) affect employees’ health behaviors. In the creation of the physical environment, designing electronic versions of “Sit Less and Move More” brochures (Lin et al., 2018) and setting up signs (Watanabe et al., 2018) and posters to encourage movement (Duncan et al., 2015) can encourage users to increase their physical activity. Optimizing the artistic design of stairs and stairwells (Colenberg et al., 2022) can encourage employees to climb stairs more often. Planning bicycle storage and bicycle lanes (Watanabe et al., 2018) promoting employees’ engagement in cycling sports, designing ergonomic standing fitness stations (Franke and Nadler, 2021), maintaining a certain distance between workstations (Jens and Gregg, 2021), and increasing flexible office locations (Engelen et al., 2019) can reduce employees’ sitting for long periods. Placing healthy foods and beverages in prominent positions (Colenberg et al., 2022) promotes healthy and nutritious eating among employees. Designing seating areas (Candido et al., 2019) and the addition of biophilia and green design (Candido et al., 2019) are conducive to the office community implementing pressure-reduction activities. Designing atriums and larger daylight channels (Candido et al., 2019) provides employees with more natural light. Regarding the social environment, employee behaviors are affected by interpersonal relationships, such as with family, friends, and colleagues (Pahn and Yang, 2021) on the one hand and are intervened by the management system of the company and the organization (Maphong et al., 2022) on the other. These strategies will encourage employees to reduce sedentary activity and strengthen physical activity, such as the elimination of desk ownership and the implementation of a workstation rotation system (Candido et al., 2019), the prohibition of bringing food into the workstation, the encouragement of walking out to eat (De Cocker et al., 2015), the use of online mini programs to conduct employee clock-in activities, and regular step log feedback (Zhang et al., 2015; Iyengar et al., 2019). In addition, the company’s provision of nutritional counseling (Matson-Koffman et al., 2005) and mental health services (Burgess et al., 2019) also plays a role in promoting healthy eating and decompression in office groups. Therefore, under the challenge of returning to office after the pandemic, combined with the trend in office work toward smart work, work content in the new era imposes new development requirements on office space design and creates a collective need for healthy, comfortable, and intelligent office environments.

In summary, the construction of a healthy office space for office groups to promote individual health by promoting healthy behaviors is urgently needed for research and implementation. The objectives of this study are as follows: (1) To explore the factors affecting the health behaviors of an office group based on the theory of planned behavior theoretical framework, (2) To study the practical effectiveness of relevant strategies in promoting office health behaviors at the architectural design level, (3) to study the important role of gender in the health behavior orientation of the office group by incorporating gender factors into the research framework and consider the differential impacts of health behavior interventions at the individual and group levels, and (4) to emphasize the differentiated management and design of the built environment and provide optimization suggestions for the scoring system of healthy office environments and the WELL Healthy Building Standard. This paper attempts to find new ways to supplement the relevant studies on architectural strategies and increase the number of study cases of office groups to provide new research ideas and methods for scholars in the field of architectural design.

This paper will be studied and discussed in the following sections. First, the background and significance of this research are expounded in the introduction, namely, the importance of health and healthy behaviors, the status quo of relevant research on healthy buildings and office groups, and the urgency of healthy office space creation. Second, the literature review section reviews the role of gender in various fields, especially in the field of built environment, and the characteristics of office groups and the theory of planned behavior and its research status will be summarized. The Methods section describes the main research steps, research objects, data collection, and analysis methods. In addition, the differences in gender characteristics, formation mechanism of health behaviors, and effectiveness of architectural strategies are analyzed and expounded in the results section. It was found that male and female employees had significant differences in individual characteristics, implementation of health behaviors, and degree of intervention in different architectural strategies. The Discussion section discusses the findings, implications, shortcomings, and future directions. Finally, the research findings and significance are summarized in the conclusion, and new research methods, how to solve the literature gap, and future research direction and focus are summarized and discussed.

## 2. Literature review

### 2.1. Review of the literature on gender

Research in recent years has begun to focus on key drivers and differences of gender in various fields. At the cognitive level, it has been found that men and women have different awareness of, attitudes about, and reactions to health risks (Yoo and Baek, 2019), humor (Hofmann et al., 2020), risk-taking (Jetter and Walker, 2020), death, and pro-environmental positions (Wang et al., 2022), and exhibit gender stereotypes (Mertens et al., 2021). In terms of preference choices, studies have shown that men and women choose different types of digital games (Lange et al., 2021), toys (Davis and Hines, 2020), internet usage patterns (Kovacevic and Kascelan, 2020), eating behaviors (Mertens et al., 2021), and so on. Gender differences persist in preferences for a long time (Baudin and Hiller, 2019). In mental health studies, female administrators are at a greater risk than males for sleep disturbances (Marelli et al., 2020), female students are more likely to develop mental illnesses because of environmental influences (Marelli et al., 2020), and women have been observed to have higher levels of loneliness in older age groups (Cohen-Mansfield et al., 2016). In terms of research to promote physical activity, most studies have shown that men report higher levels of physical activity than women, whether older adults (Keadle et al., 2016), adults (Antunes et al., 2020), or children (Leech et al., 2014).

Previous studies reported differences in the prevalence of office-related symptoms between male and female office groups. The two groups had different perceptions of stress (Stigsdotter et al., 2010) and recovery rates (Wang et al., 2007; Weekes et al., 2008), and more professional women experienced greater stress than men (Stigsdotter et al., 2010; Lottrup et al., 2013). In a study on subjective well-being, women’s job satisfaction was progressively lower than that of men (Batz-Barbarich et al., 2018). Different circadian rhythms also led to different sleeping behaviors and patterns (Duffy and Czeisler, 2009). Compared to women, men slept later and awakened later (Duffy et al., 2011). Women are generally more overweight and obese than men (Nogueira et al., 2020). Globally, the proportion of women who were inactive and lacked physical activity was much larger than that of men, and few women reached the internationally recommended level of physical activity (Adlakha and Parra, 2020). In addition, the proportion of female staff who reported sick building syndromes, such as problems with their eyes, nose, or neck, skin diseases, headaches (Brasche et al., 2001; Aries et al., 2010), and multisite musculoskeletal pain (Treaster and Burr, 2004; Hooftman et al., 2009), was significantly higher than that of male staff. Based on the above health issues, personal health behaviors are not only related to environmental and social factors but they should also consider the structural influence of gender.

Different built environments have different impacts on women and men and to different degrees. In addition to gender differences caused by biological factors, individual gender role attributes, behaviors and activities under social construction also play a very important role. For example, studies have shown that women are more focused on cleanliness, esthetics, and safety, and prefer separate event Spaces and nearby recreational facilities. Men, on the other hand, attach importance to the connectivity of facilities and streets in social activities, so that they can reach their destinations and achieve their goals more quickly (Valson and Kutty, 2018).

Furthermore, in the same built environment, the perceptions and responses of different user groups are often significantly different (Ma and Dill, 2016; Brookfield et al., 2020), and different interventions mean optimizing the environment to promote health behaviors, resulting in a deviation in effects (Xie et al., 2021). The social constructed differences between girls/women and boys/men contribute significantly to the observed differences (Valson and Kutty, 2018). The gender difference can be shown as follows: in terms of promoting sports activities, increases in bicycle paths (Mitra and Nash, 2019), public transport stations (Wang et al., 2022), and more vegetation coverage (Nawrath et al., 2019) could help to improve the probability that women would ride bicycles. The increase in infrastructure, such as commercial infrastructure (Wang et al., 2022), and the improvement in utilization rates for mixed land functionality (Maghelal et al., 2022) could help convince men to ride bicycles. However, the better the built environment is for traffic accessibility (Mertens et al., 2021) and sports facilities (Higuera-Mendieta et al., 2021), the better the increased riding behavior of the two groups, in turn promoting their physical activity levels. In addition, the study found that a lack of walking infrastructure (Adlakha and Parra, 2020), traffic congestion, and poor street connectivity (Gao et al., 2022) were often obstacles for women’s walking trips. There were also gender differences in sports preferences between the two groups. Women preferred non-contact sports and recreational activities, such as walking and dancing, whereas men preferred team and contact sports, such as basketball and football. Therefore, different sports venues demonstrated different effects after promotion (Nogueira et al., 2020). In terms of mental regulation, eliminating graffiti on the walls and demolishing solid walls blocking the line of sight could improve women’s sense of uneasiness (Navarrete-Hernandez et al., 2021), and an environment with easy access to shops, public transportation, entertainment facilities, and well maintained and safe sidewalks helped women alleviate their depression or fears (Koohsari et al., 2019). Maximizing the use of natural elements and increasing physical contact and visual access to green plants significantly reduced and relieved men’s psychological stress (Lottrup et al., 2013). Increasing outdoor infrastructure and reducing noise pollution had positive effects on the mental health of both groups (Shen et al., 2021). The thermal environment played an important role in ensuring healthy sleep. Men’s sleep quality was generally better in a lower-temperature environment, while women preferred a warm sleep environment (Irshad et al., 2019). In terms of regulating individual thermal comfort, women were more sensitive to temperature fluctuations than men. Better personal control, a larger thermal environment regulation area, and an air ventilation mode could improve the satisfaction rate of women (Indraganti and Humphreys, 2021). In conclusion, different groups using the same building space should benefit equally from the planned intervening measures; therefore, the above intervening means should consider the needs of different target groups and influences, such as gender differences.

As society and civilization progress, scholars from various fields have begun to explore the different subjective feelings and actual needs of men and women based on gender differences, including gender stratification analysis of special groups such as children (Indraganti and Humphreys, 2021), teenagers (Brookfield et al., 2020; Maghelal et al., 2022), and older adults (Jia et al., 2018; Koohsari et al., 2019; Namgung et al., 2019; Yu et al., 2021). Similarly, against the background of the increasing number of female staff members, many studies have discussed how to promote staff health more effectively according to gender differences (Lee et al., 2018; Kim and Bluyssen, 2020). However, most of these studies focused on the optimization design of indoor environment quality (Awada and Srour, 2018), greening (Awada and Srour, 2018), hot and cold environments (Liu et al., 2018; Irshad et al., 2019; Indraganti and Humphreys, 2021), and ventilation systems (Yang et al., 2020); however, they have never commenced from the overall strategy system of office space.

### 2.2. Review of the literature on the theory of planned behavior

**Figure 1:**
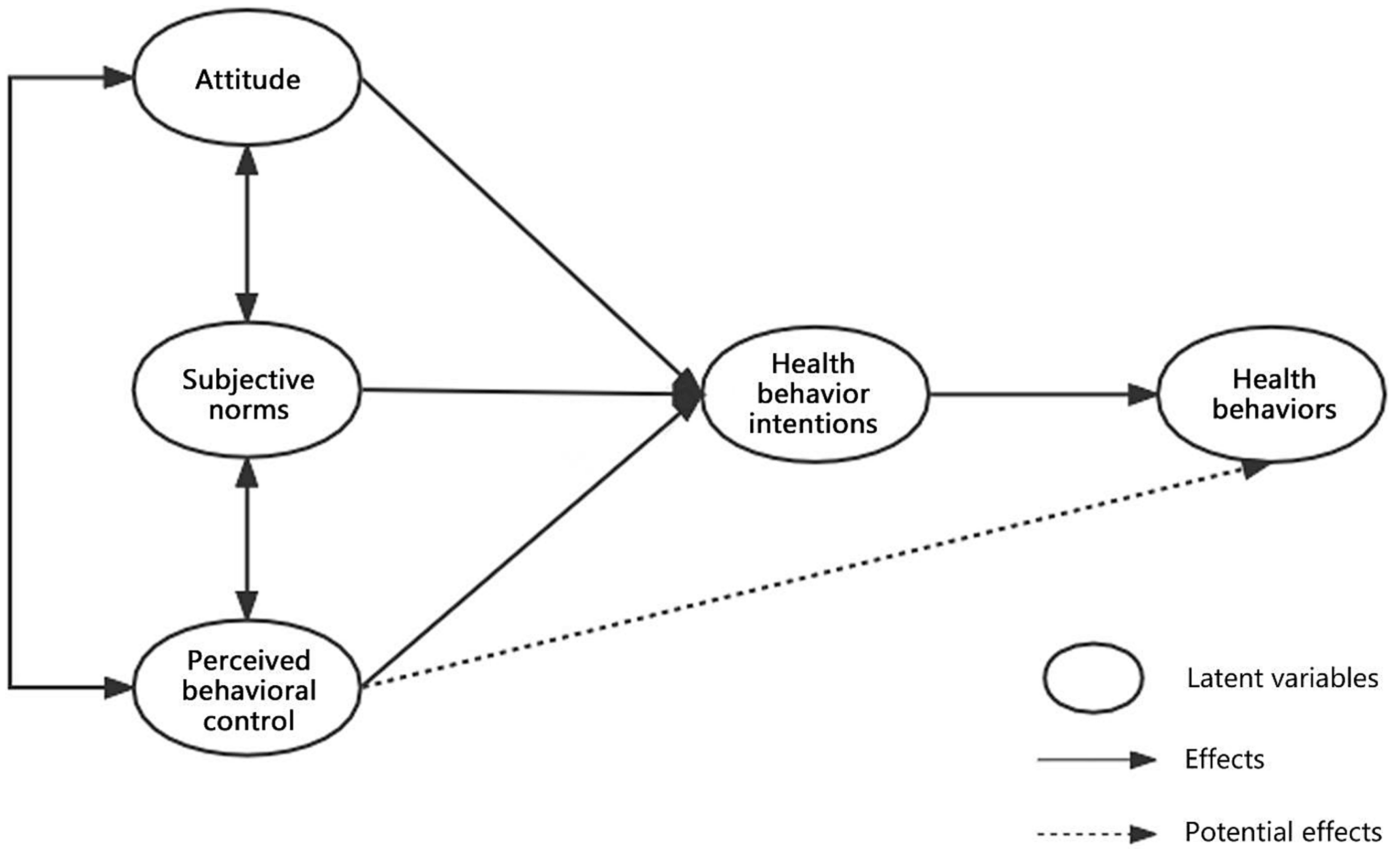
The TPB model.

In 1985, the theory of planned behavior (TPB), which evolved from the theory of rational behavior, was proposed in the field of social psychology. Currently, it is one of the most widely applied theories in health behavior research (Guo, 2022). This theory is mainly used to explore the mechanisms of the psychological factors that influence people’s behaviors. It includes five variable elements and their interrelations. Attitude refers to an individual’s positive or negative attitude toward a behavior. Subjective norms refer to the social stress that an individual feels when they undertake specific actions, such as the suggestions of relatives, friends, and colleagues and the regulations of the company system. Perceived behavioral control refers to an individual’s cognition of the difficulty of executing a specific behavior, including whether they have mastered resource conditions and opportunities. Behavioral intention refers to an individual’s thoughts and inclinations about undertaking certain actions. Actual behavior refers to the actions implemented by an individual. According to this theory, behavioral intentions directly influence or determine the generation of behaviors, but behavioral intentions are in turn influenced by attitudes, subjective norms, and perceived behavioral control. In other words, the more positive the attitude, the greater the support of important others, and the stronger the perceived behavior control, the stronger the behavioral intentions, and the easier it will be to perform the behaviors. The theoretical framework of TPB is shown in Figure 1.

Since the TPB was proposed, it has been widely used in many research fields to explore the causes of specific behavioral mechanisms, including drivers speeding and other violations in the field of safety (Warner and Aberg, 2006; Forward, 2009), energy saving and waste reduction in the field of energy conservation and environmental protection (Liu et al., 2020; Farani et al., 2021; Wang et al., 2021; Teoh et al., 2022), public transport travel in the field of transportation planning (Fu and Juan, 2017; Ng and Phung, 2021), production, consumption, and entrepreneurship in the field of the economy (He and Veronesi, 2017; Scalco et al., 2017; Ataei et al., 2021), law-abiding and compliance in the field of social morality (Cooper, 2017; Wang et al., 2022), and farmers’ cultural bias and adaptive behaviors in the field of agriculture (Karimi and Ataei, 2022). In recent years, scholars have also used this theory as a theoretical framework to promote specific health behaviors, such as healthy eating (Prestwich et al., 2014), physical exercise (Gourlan et al., 2016), and ending addictive behaviors such as smoking (Lareyre et al., 2021), health care behaviors (Sniehotta et al., 2014), and oral hygiene behaviors (Burns, 2009). However, in previous studies, TPB was mostly used to study the influencing factors of certain health behaviors, and systematic and diversified health behavior research has been insufficient. Simultaneously, such research has focused on the psychological causes of behaviors but has not yet been applied to the field of actual architectural design. Therefore, this study attempts to use the TPB theory to study the health behaviors of office groups to promote the development of healthy buildings.

The original TPB can be divided into two levels: the influence of behavioral intentions on shaping actual behaviors, and the factors affecting behavioral intentions, including attitudes, subjective norms, and perceptual behavioral control. The purpose of this study was to analyze the factors influencing the health behaviors of an office group and explore the actual effectiveness of existing building management and design strategies. Therefore, based on the original two levels, we added a third level of discussion: the factor analysis of the second level, that is, the analysis of factors affecting attitudes, subjective norms, and perceptual behavior control. This study attempted to integrate architectural strategies into model construction (see model construction below for details).

### 2.3. Summary

Considering the above research status, there have been few studies on a more systematic variety of health behaviors in office groups and there have been insufficient studies exploring the practical effectiveness of WELL health promotion intervention strategies. Gender, as an important influencing factor for health and health behaviors, is often not explicitly addressed or explored in health promotion research on the built environment (Fisher and Makleff, 2022). Therefore, it is of important research value and significance to study the health behaviors and influencing factors of office groups in the office environment, promote the implementation of more effective building strategies, and explore the specific role of gender on the influencing path.

## 3. Materials and methods

**Figure 2:**
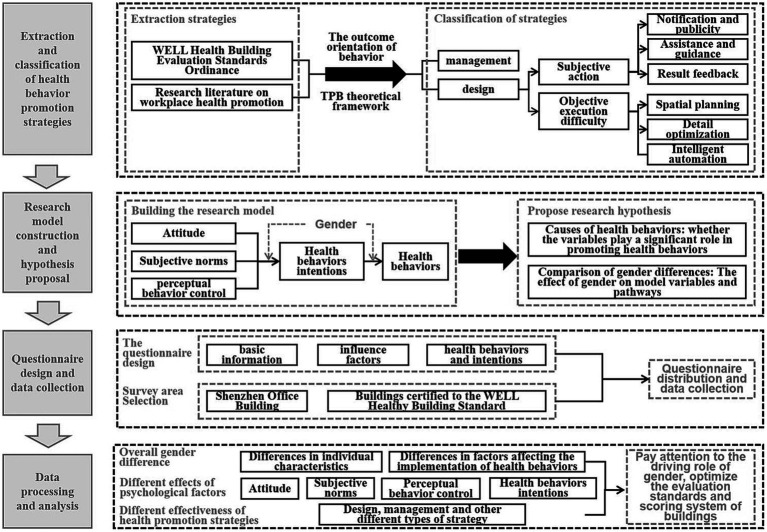
Overall research method flowchart.

The steps of this study are as follows: (1) We reviewed the regulations of the WELL Healthy Building Standard and related office environment literature and extracted and summarized strategies related to promoting healthy behaviors. (2) Strategies are classified according to the result-oriented behavior and TPB theoretical framework, which can be divided into management and design. The design strategies can be further subdivided into subcategories that affect individual subjective action and objective implementation difficulty. (3) Based on the TPB, gender elements are included in the construction of research models, and research hypotheses are proposed. (4) Questionnaires are designed according to the research model, and office groups in the selected areas are surveyed. (5) AMOS, SPSS, Excel, and other software were used to verify the feasibility of the data and research model, followed by relevant statistical analysis, structural equation analysis, analysis of variance, and so on, to explore the influencing factors and differences in health behaviors of different gender office groups. Suggestions were offered to maximize the effectiveness of the strategies. The overall research method flow is shown in Figure 2.

### 3.1. Research process

#### 3.1.1. Extracting and dividing architectural strategies

This study first reviewed the regulations of WELL building standards and related literature (see above for strategic sources) to summarize and generalize strategies related to promoting healthy behaviors. Subsequently, combined with the meaning of each variable of the TPB and the theoretical framework, the strategies were divided into management and design levels. Combined with the outcome orientation of the behaviors (i.e., deciding whether to implement health behaviors), the design strategies were divided into two: subjective action and objective execution difficulty. From the user’s subjective perspective, subjective actions can reflect the user’s subjective intentions to implement health behaviors. We can increase the user’s subjective identity through certain design means and promote the generation of more positive behavioral attitudes. However, objective execution difficulty refers to the convenience or difficulty in implementing health behaviors, which can affect users’ confidence in behavior control to a certain extent. In the strategy classification, subjective design strategies can affect attitudes to a certain extent, company management strategies can affect subjective norms, and objective design strategies of health behaviors can affect perceived behavioral control.

**Figure 3:**
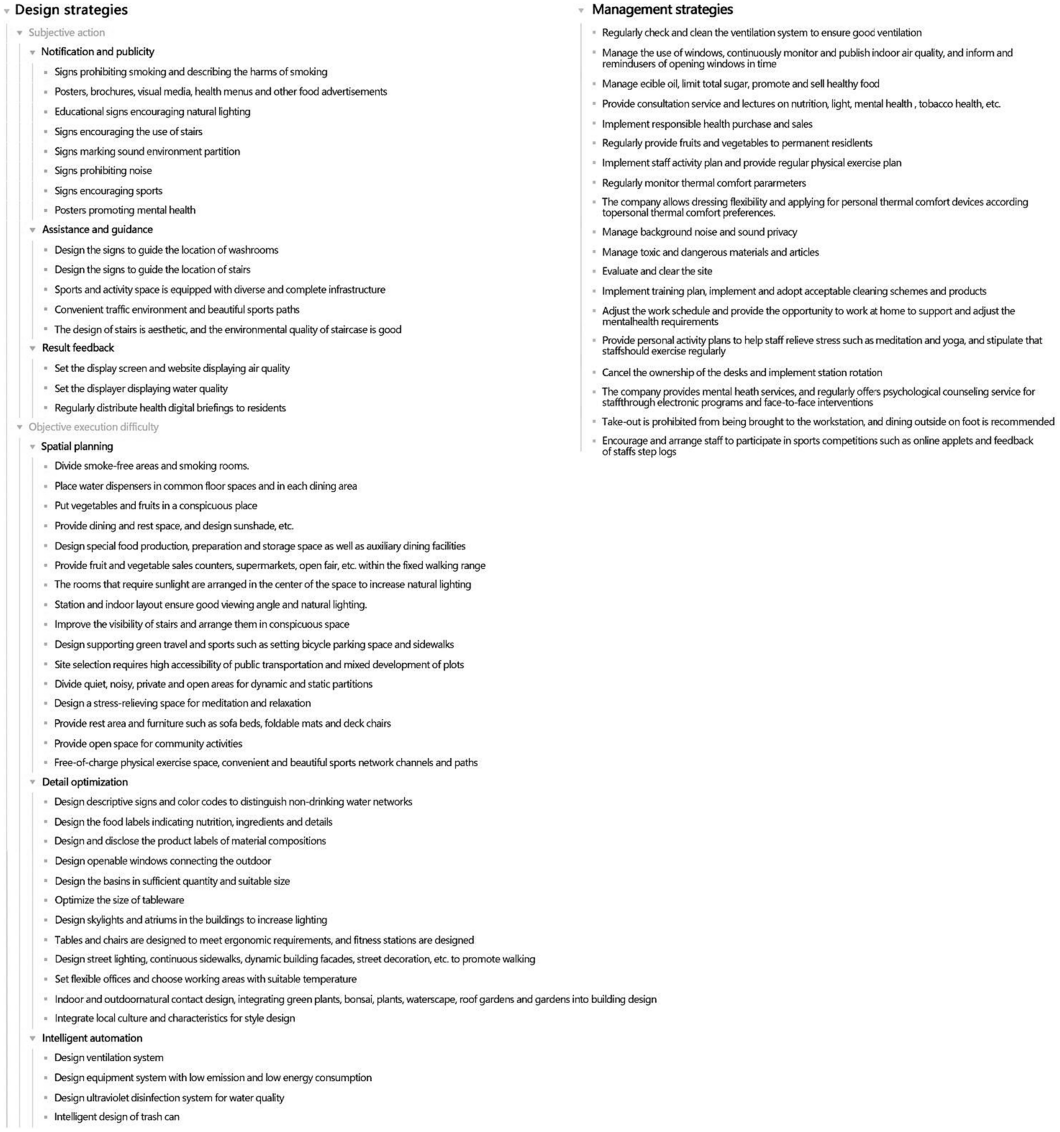
Strategies related to health behaviors and their classification.

On this basis, according to the literature sources for each strategy (see the Introduction), we further subdivided the design strategies of subjective and objective categories into subcategories: subjective action including notification and publicity (improving users’ health cognition and increasing actions by publicizing health behaviors), assistance and guidance (stimulating users’ interest in actions by creating a good environment and atmosphere), and feedback (regular feedback to the user for a period of time about the behavior of the results to achieve a long-term healthy and active lifestyle). The difficulty of objective implementation can be reduced by spatial planning (dividing objective spaces and functions), detailed optimization (humanization, naturalism, artistic enhancements, and other specific designs), and intelligent automation (adding scientific and technological elements and products). See Figure 3 for the health-related strategies and their classifications.

#### 3.1.2. Building a research model and proposing assumptions

**Figure 4:**
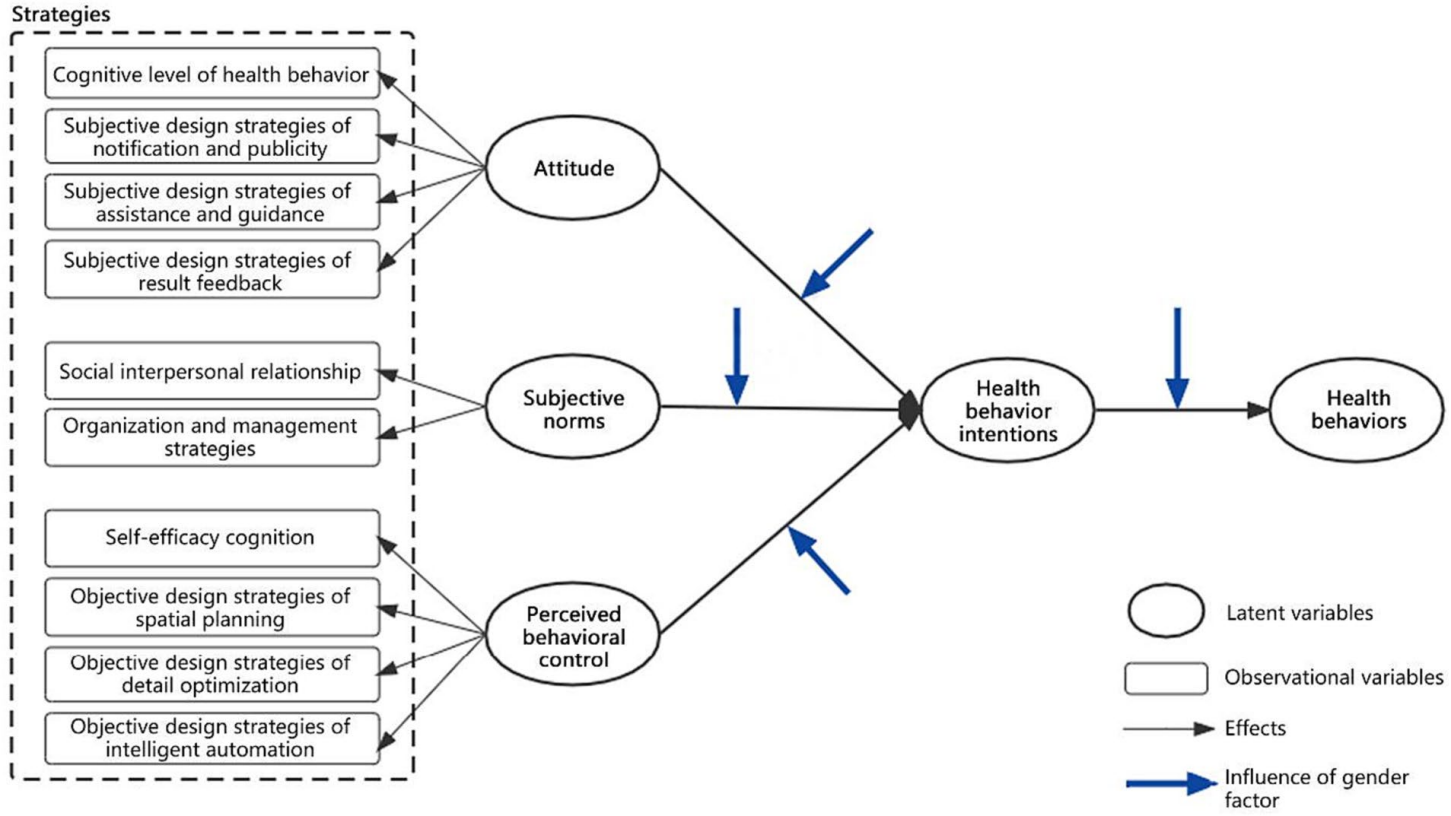
Establishment of the research model.

The TPB framework was adjusted and extended according to the content of this study. This study deletes the potential influence path of perceived behavioral control on health behaviors in the original model, and integrates architectural strategy categories into the research model, that is, each strategy category can be used as an observation variable to influence the corresponding potential variables. The role of gender in the research mechanism and each influence path are added. Simultaneously, based on the research model (see Figure 4), two research hypotheses are proposed (see Table 1): the causes of health behaviors and the effects of gender differences on the influence paths of health behaviors and the comparative direction of gender differences, which are expressed as H1 and H2.

**Table 1:** Summary of hypotheses.

SN	Research hypotheses
H1	Attitudes, subjective norms, and perceived behavioral control of the office groups have a significant, positive correlation with the health behavior intentions and subsequently affect the health behaviors.
H1a	The attitudes of office staff (cognition level of health behaviors, notification and publicity, assistance and guidance, and result feedback) have a significant correlation with their health behavior intentions and subsequently affect health behaviors.
H1b	The subjective norms of office building environment (social interpersonal relationships, organization, and management strategies) have a significant correlation with their health behavior intentions and subsequently affect health behaviors.
H1c	The perceived behavioral control (self-efficacy cognition, spatial planning, detail optimization, and intelligent automation) of an office building environment has a significant correlation with workers’ health behavior intentions and subsequently affects health behaviors.
H1d	The health behavior intentions of the office building environment have a significant correlation with health behaviors.
H2	Gender has some influences on the variables and paths of the research model.
H2a	There are significant gender differences among the attitudes, subjective norms, perceived behavioral control, behavior intentions and behaviors of the office groups.
H2b	There are gender differences in the influence of the attitudes of the office groups (health behavior cognition level, notification and publicity, assistance and guidance, and result feedback) on health behavior intentions and health behaviors.
H2c	There are gender differences in the influence of the subjective norms of the office groups (social interpersonal relationships, organization, and management strategies) on health behavior intentions and health behaviors.
H2d	There are gender differences in the influence of perceived behavioral control of the office groups (self-efficacy cognition, spatial planning, detail optimization, and intelligent automation) on health behavior intentions and health behaviors.
H2e	There are gender differences in the influence of health behavior intentions of the office groups on health behaviors.

The focus of this study is to explore various factors that affect health behaviors, especially to test the effects of different building strategies and whether there are significant differences between men and women in the effects of these factors on office group behaviors. Therefore, this study conducted a comparative analysis of several groups by using multiple structural equation models. On the one hand, this study divides the whole sample into male and female groups to build a model framework for the two groups to facilitate comparison. On the other hand, it divides the first-and second-level variables as well as the corresponding two-level variable models. The former includes attitudes, subjective behavior norms, perceived behavioral control, health behavior intentions, and health behaviors, whereas the latter includes subjective cognition, notification and publicity, assistance and guidance, result feedback, social relationships, organization and management, self-efficacy cognition, spatial planning, detail optimization, intelligent automation, health behavior intentions, and health behaviors. Two levels of models are used to explore the different effects of factors and specific strategies that influence the health behaviors of office groups.

### 3.2. Participants

**Figure 5:**
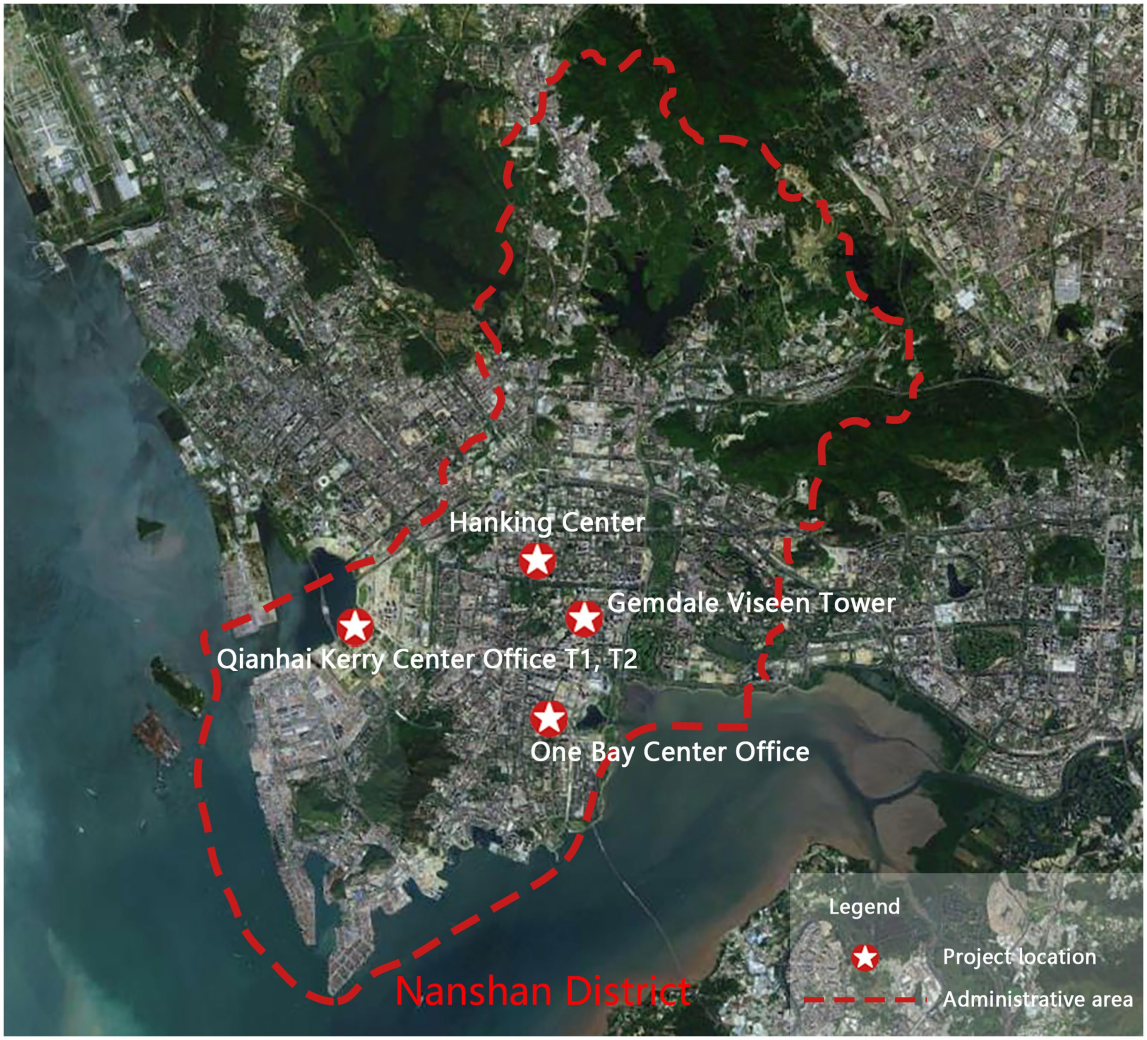
Location distribution map of the selected office buildings.

As a special economic zone in China, Shenzhen is a national economic center and an international city. Nanshan District is a high-tech industrial base in Shenzhen with a large working population and many office buildings. Office staff members have a fast-paced life and great work pressure; therefore, there is a broad range of typical population samples in this area. Simultaneously, the practical experience of healthy buildings in Shenzhen leads the entire country. As of August 2022, there were 151 WELL-certified projects in Shenzhen, China. In recent years, healthy buildings in Nanshan District have shown a vigorous development trend. Four WELL healthy office buildings in this area were selected for the survey. See Figure 5 and Table 2 for basic information on the selected office buildings.

**Table 2:** Basic information about the selected office buildings.

Name of building	Certified area of the project (m^2^)	Number of floors (floors)	WELL certification type	Certification date	Number of questionnaires (copies)
One Shenzhen Bay	66,850.99	72	WELL V1 Gold	August 21, 2019	127
T1, T2, Qianhai Kerry Center	133,272.7	29	WELL V1 Gold	March 23, 2021	150
Viseen International Center	226,897.8	48	WELL V2 Core Approved	June 17, 2021	170
Hanking Center Tower	167,001.3	61	WELL HSR Certification	February 22, 2021	150

**Figure 6:**
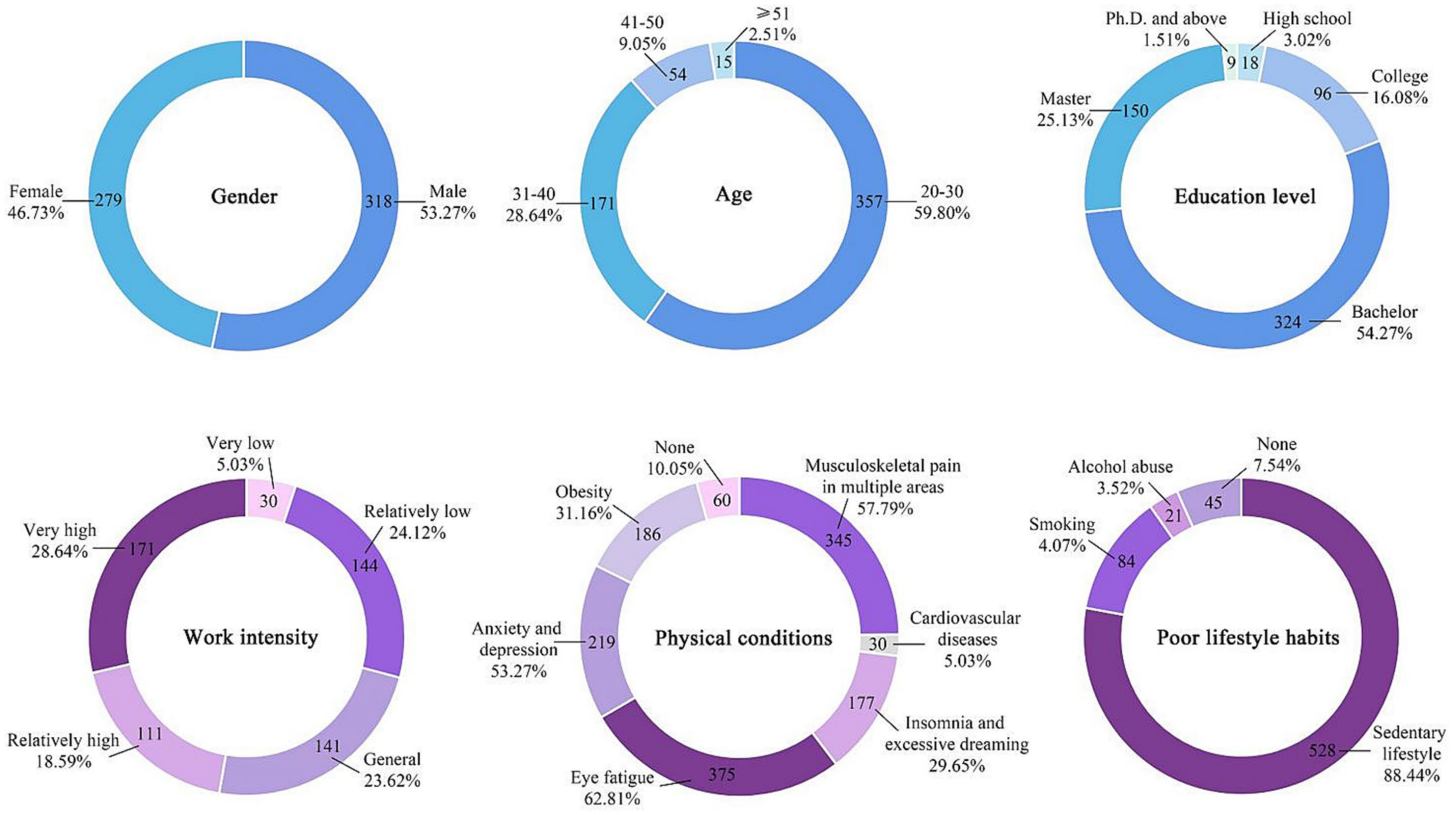
Sample structure characteristics of user questionnaires.

**Figure 7:**
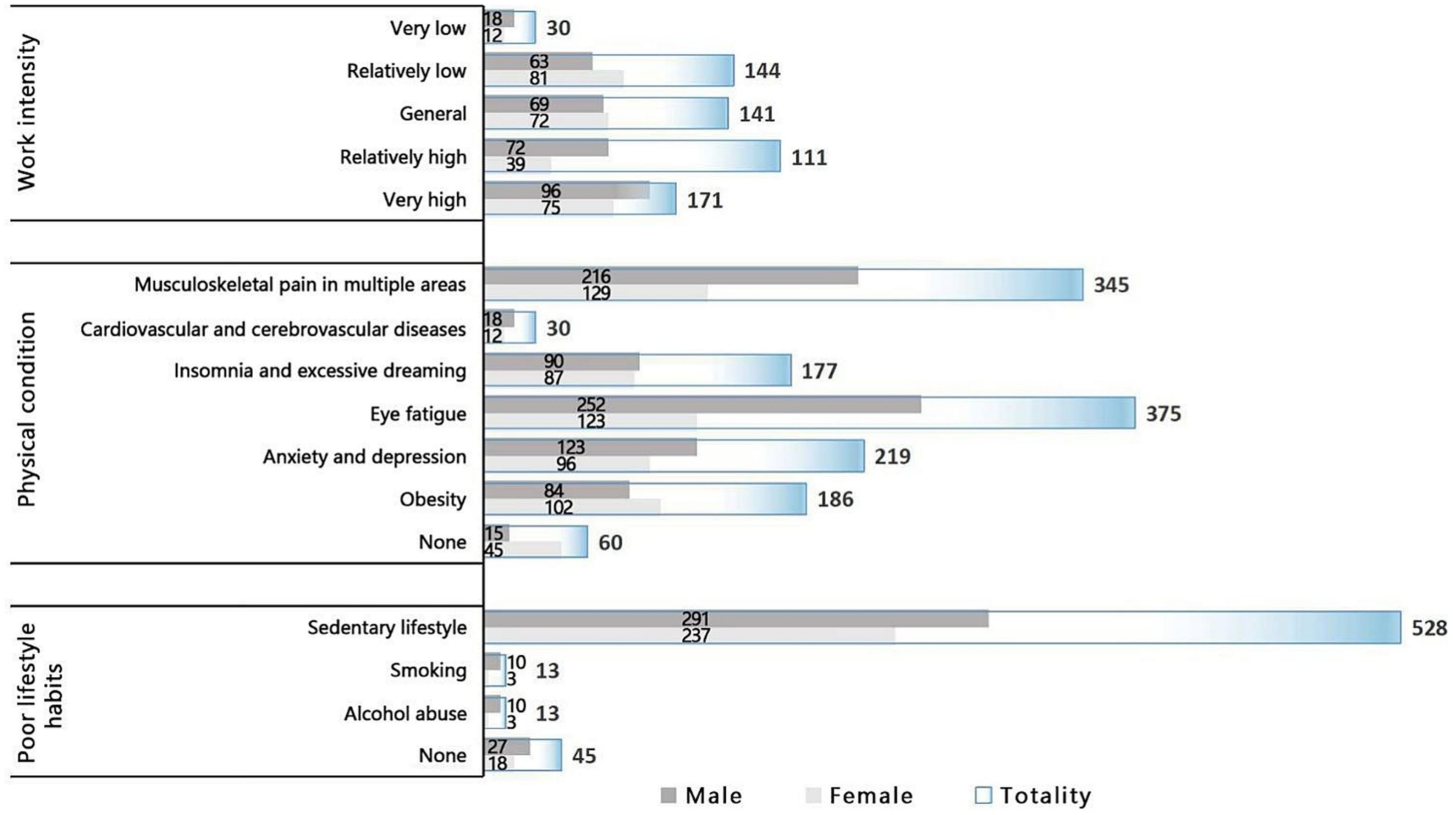
Sample structure characteristics of gender-based user questionnaires.

In this study, user data were collected using a questionnaire survey. The questionnaire design was implemented according to the above research framework, which was mainly established in three aspects: basic information, influencing factors, and health behaviors and intentions. The questionnaire design is detailed in Appendix A. Basic information included gender, work intensity, physical conditions, and bad living habits. The influencing factors corresponded to the variables of the research model, namely attitudes, subjective norms, and perceptual behavior control. Work intensity, influencing factors, health behaviors, and intentions were evaluated using a five-point Likert scale, and the participants were required to score 1–5 points quantitatively according to their situation. In this study, high-quality data were recovered by inviting office groups to complete questionnaires in the survey area. Finally, 597 valid questionnaires were collected from December 10, 2021, to January 10, 2022, including 318 valid questionnaires for men and 279 valid questionnaires for women. See Figures 6, 7 for the basic information characteristics of the respondents.

### 3.3. Data analysis

On the one hand, this study will conduct descriptive statistical analysis of the collected data, including the mean, *t*-test, and variance analysis, to explore the differences in the basic characteristics of the male and female office groups. We analyzed the different influencing factors and degrees of influence on the health behaviors of the two groups based on the research model framework. On the other hand, this study mainly used the method of constructing structural equation models for data analysis. Compared to traditional statistical analysis methods, this method is more suitable for processing large samples and can improve the scientific nature of the method through reliability and validity, fitting analysis, and purification error. Structural equation modeling can also better analyze latent variables that are difficult to measure accurately and directly, such as attitudes, subjective norms, and perceived behavioral control, and evaluate the correlation relationships between multiple complex variables and indicators simultaneously.

## 4. Results

Two structural equation models are constructed based on the research framework: a first-level variable model (to explore the factors affecting health behaviors) and a second-level variable model (focusing on the impacts of promotion strategies on health behaviors). The male and female groups were compared at each level of the variable model.

### 4.1. Analysis of differences in overall gender characteristics

The analysis of average values and gender *t*-tests indicated that there were differences in individual characteristics between male and female office groups. First, men’s work intensity and stress were slightly higher than women’s (the average values of work intensity were 3.52 and 3.30, respectively, and the sig value was <0.05). Second, in terms of physical condition, the high incidence of diseases between the two groups was slightly different (the sig value of most diseases was <0.05), and men were more prone to eye diseases. This finding proves that men had increased work and eye stress. Although women were more prone to musculoskeletal diseases, the average value was still lower than that of men. The average incidence of insomnia and obesity in women was higher than that in men. This outcome might have occurred because there were no clear definitions for the diseases in the questionnaire of this study; therefore, there were differences between individual cognition and common-sense standards. For example, most women are sensitive to weight and believe that being overweight is indicative of obesity. Furthermore, the most common bad habits of the two groups were sedentary (the average values were 0.92 and 0.85, respectively, and the sig value was <0.05), and its severity was far greater than that of smoking and drinking. This finding showed that the research groups were mostly intellectual workers and they were sedentary on most working days. Based on the company’s management system, office groups consisting of high-level intellectuals did not frequently consume alcohol or smoke.

Conversely, there were gender differences in the implementation of health behaviors, which affected the effectiveness of the model variables. There was no significant difference among gender, attitude, and observational variables (sig value >0.05), but there was a significant difference with other variables (sig value <0.01). This indicated that different gender preferences and characteristics determined the effectiveness of other variables to some extent, while acquired environmental changes could significantly affect attitudes. Compared with men, women’s acceptance of all variables, especially the optimization strategies of healthy buildings, was higher, and this might depend on women’s physiological characteristics. Women were more sensitive to the surrounding environment, so they were more susceptible to the environment and developed corresponding health behaviors and intentions. In summary, H2a is tenable.

### 4.2. Influencing factors and degree of health behaviors

The first-level variable model of this study mainly discussed the factors that affect the health behaviors of office groups, namely, the relationships among the variables of attitudes, subjective norms, perceived behavioral control, health behaviors, and intentions. To ensure reliability, this study tested the reliability and validity of the data using AMOS software. It mainly includes combination reliability (CR) and average variance extracted (AVE). Generally, the applicable scope of CR should be ≥0.5 (preferably >0.6), and AVE should be ≥0.5. After analysis, the other variables were at good levels, except that the AVE of health behaviors was relatively low (0.308 for men and 0.442 for women). Overall, the first-level variable model basically agreed with the reliability and validity level, and the convergence of the model was good, so it could be analyzed in the next step.

**Figure 8:**
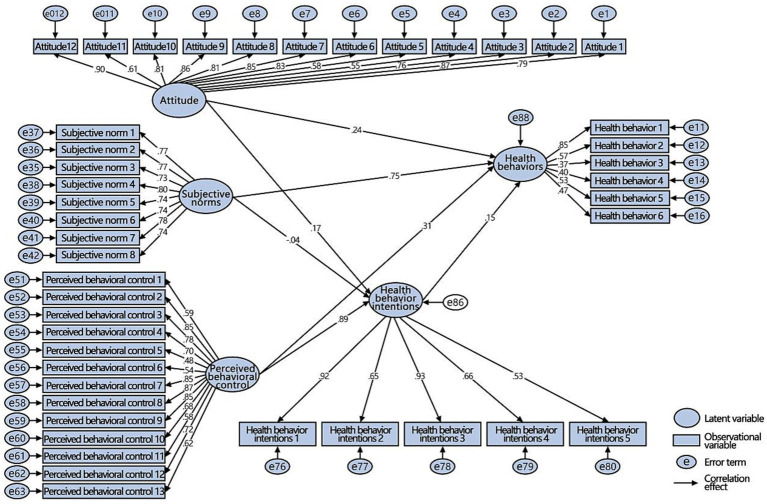
SEM of the first-level variable for men.

In the first-level variable model for men (Figure 8), attitudes and perceived behavioral control had significant positive effects on health behaviors and intentions (the *p*-values were significant on both sides at 0.001), assuming that H1a and H1c were tenable. Although subjective norms had a significant positive effect on health behaviors (the *p*-values were significant on both sides at 0.001), they had an insignificant negative effect on intentions (*p* = 0.324), assuming that H1b was tenable. Health behavior intentions also had a significant positive effect on health behaviors (the *p*-values were significant on both sides at 0.001), assuming that H1d was tenable.

**Figure 9:**
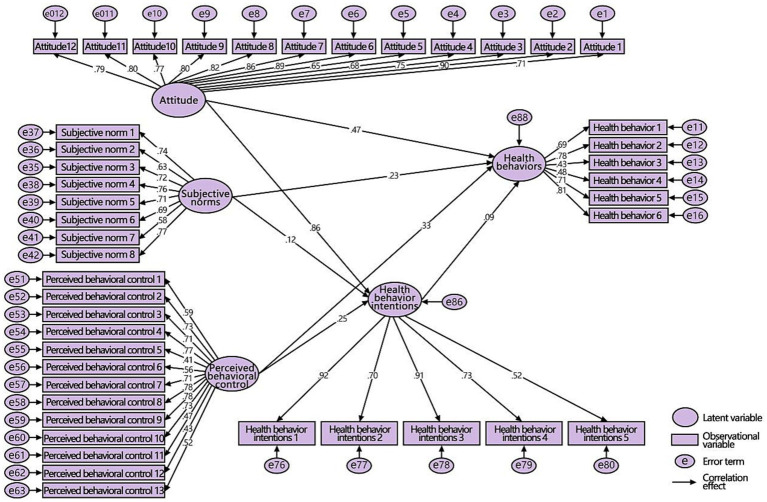
SEM of the first-level variable for women.

According to the first-level variable model for women (Figure 9), all paths showed a significant, positive relationship (the *p*-values were significant on both sides at 0.001 and 0.01), except that the *p* value of the effect of health behavior intentions on health behaviors was 0.052 and slightly higher than 0.05, assuming that H1a, H1b, and H1c were tenable, and H1d was not tenable.

Through a comprehensive comparative analysis of the first-level variable models of the two groups, it was found that the path coefficient of the female group was higher than that of the male group, and the subjective norms and health behavior intentions had different effects on their behaviors; thus, H2 was tenable. See Table 3 for data analysis and corresponding hypothesis verification.

**Table 3:** Significance analysis and hypothesis verification of the first-level variable model.

Research hypotheses		Men					Women				
		Standardization coefficient	Standard error (S.E.)	Critical ratio (C.R.)	Utility value (P)	Result	Standardization coefficient	Standard error (S.E.)	Critical ratio (C.R.)	Utility value (P)	Result
H1a	Attitude → Health behavior intentions	0.166	0.049	3.373	***	Tenable	0.858	0.063	13.612	***	Tenable
	Attitude → Health behaviors	0.235	0.025	9.478	***		0.470	0.059	7.993	***	
H1b	Subjective norms → Health behavior intentions	−0.041	0.041	−0.985	0.324	Partially tenable	0.118	0.041	2.892	**	Tenable
	Subjective norms → Health behaviors	0.750	0.041	18.189	***		0.233	0.029	7.979	***	
H1c	Perceived behavioral control → Health behavior intentions	0.890	0.090	9.881	***	Tenable	0.245	0.062	3.923	***	Tenable
	Perceptual behavior control → Health behaviors	0.309	0.044	6.972	***		0.331	0.048	6.935	***	
H1d	Health behavior intentions → Health behaviors	0.151	0.031	4.926	***	Tenable	0.088	0.045	1.947	0.052	Untenable

H1 is partially tenable, and H2 is tenable.

**Significant at 0.01 (both sides); ***Significant at 0.001 (both sides).

### 4.3. Effectiveness of WELL healthy building strategies

Compared to the first-level variable model, the second-level variable model was more complex. It could further analyze the latent variables in the first-level variable model, mainly focusing on the effectiveness of healthy building strategies. The reliability and validity analysis was generally consistent with those of the first-level variable model. In other words, the AVE value of health behaviors was low (0.217 for men and 0.336 for women), but the second-level variable model was generally consistent with reliability and validity levels.

**Figure 10:**
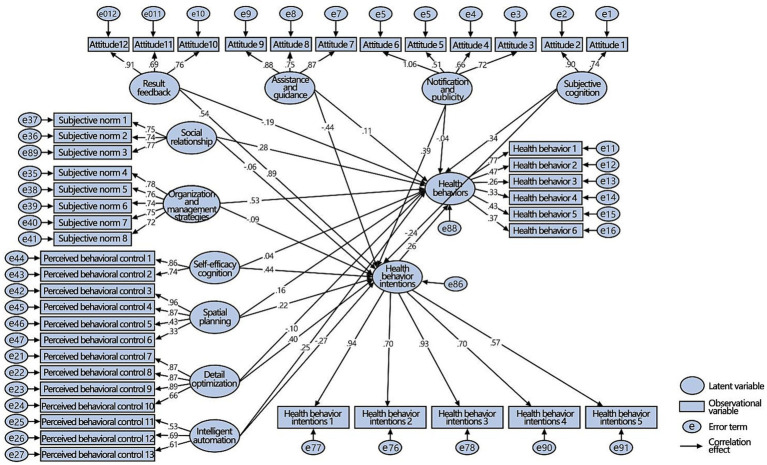
SEM of the second-level variable for men.

The analysis of the second-level variable model for men showed (Figure 10) that among 21 paths, 12 paths were significantly positive, six were significantly negative, and three were not significant. Spatial planning design strategies had a significant positive effect on health behaviors and intentions. Subjective cognitive level, assistance and guidance, social interpersonal relationships, organization and management strategies, and intelligent automation strategies had significant positive effects on health behaviors. Result feedback, notification and publicity, self-efficacy cognition, spatial planning, and detail optimization only had a significant positive effect on health behavioral intentions. Result feedback and detailed optimization strategies played a significant role in health behaviors, but the direction of action was the opposite. Social relationships had no significant effect on health behaviors or intentions. There was a significant positive correlation between health behavioral intentions and health behaviors. In summary, H1a, H1b, and H1c were partially tenable, whereas H1d was completely tenable.

**Figure 11:**
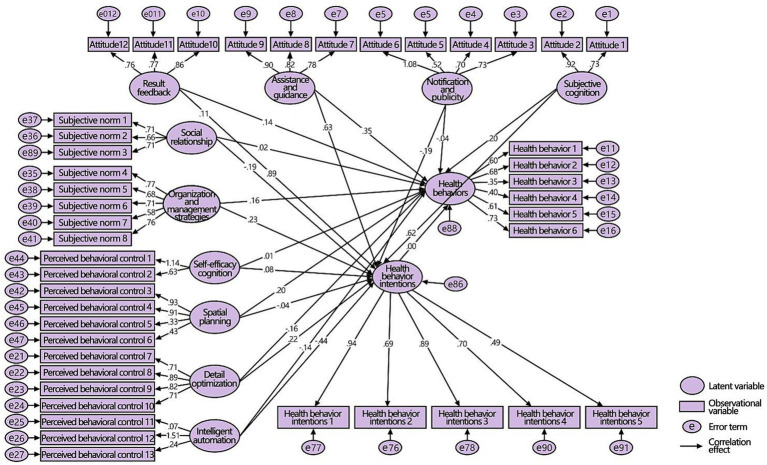
SEM of the second-level variable for women.

Compared to men, there were also 12 significant positive paths in the second-level variable model for women (Figure 11), but there were fewer negative paths and more insignificant paths. There were three significant paths and seven insignificant paths. Result feedback, assistance and guidance, subjective cognition, organization and management, and detail optimization had significant positive effects on health behaviors and intentions. Notification, publicity, and social relationships had significant negative effects on behavioral intentions but did not significantly affect behaviors. Self-efficacy cognition and spatial planning only had significant positive effects on behavioral intentions and behaviors, whereas health behavior intentions had no significant effect on health behaviors. In summary, H1a, H1b, and H1c were partially tenable, whereas H1d was not tenable.

In the second-level variable models of the two groups, all the latent variables and their observational variables had different impacts on different users, assuming that H2 was tenable. See Table 4 for data analysis and corresponding hypothesis verification.

**Table 4:** Significance analysis and hypothesis verification of the second-level variable model.

Research hypotheses		Men					Women				
		Standardization coefficient	Standard error (S.E.)	Critical ratio (C.R.)	Utility value (P)	Result	Standardization coefficient	Standard error (S.E.)	Critical ratio (C.R.)	Utility value (P)	Result
H1a	Subjective cognitive level → Health behavior intentions	−0.239	0.052	−4.571	***	Partially tenable	0.619	0.059	10.477	***	Partially tenable
	Subjective cognitive level → Health behaviors	0.341	0.035	9.680	***		0.196	0.055	3.526	***	
	Design strategies of notification and publicity → Health behavior intentions	0.394	0.047	8.350	***		−0.190	0.033	−5.802	***	
	Design strategies of notification and publicity → Health behaviors	−0.040	0.028	−1.445	0.148		−0.035	0.021	−1.653	0.098	
	Design strategies of assistance and guidance → Health behavior intentions	−0.436	0.043	−10.028	***		0.631	0.050	12.612	***	
	Design strategies of assistance and guidance → Health behaviors	0.107	0.032	3.319	***		0.351	0.060	5.806	***	
	Design strategies of result feedback → Health behavior intentions	0.538	0.058	9.266	***		0.114	0.037	3.048	**	
	Design strategies of result feedback → Health behaviors	−0.190	0.041	−4.579	***		0.137	0.026	5.318	***	
	Social interpersonal relationship → Health behavior intentions	−0.060	0.040	−1.526	0.127		−0.188	0.045	−4.171	***	
	Social interpersonal relationship → Health behaviors	0.283	0.025	11.220	***		0.021	0.028	0.745	0.456	
H1b	Organization and management strategies → Health behavior intentions	−0.094	0.035	−2.671	**	Partially tenable	0.231	0.038	6.155	***	Partially tenable
	Organization and management strategies → Health behaviors	0.528	0.032	16.699	***		0.160	0.031	5.242	***	
	Self-efficacy cognition → Health behavior intentions	0.438	0.037	11.984	***		0.078	0.024	3.259	**	
	Self-efficacy cognition → Health behaviors	0.041	0.031	1.310	0.190		0.008	0.014	0.551	0.582	
H1c	Design strategies of spatial planning → Health behavior intentions	0.218	0.042	5.152	***	Partially tenable	−0.036	0.034	−1.053	0.292	Partially tenable
	Design strategies of spatial planning → Health behaviors	0.156	0.024	6.490	***		0.201	0.026	7.876	***	
	Design strategies of detail optimization → Health behavior intentions	0.397	0.042	9.452	***		0.218	0.053	4.118	***	
	Design strategies of detail optimization → Health behaviors	−0.099	0.030	−3.304	***		0.157	0.036	4.293	***	
	Design strategies of intelligent automation → Health behavior intentions	−0.267	0.076	−3.527	***		−0.438	0.367	−1.194	0.232	
	Design strategies of intelligent automation → Health behaviors	0.252	0.048	5.254	***		−0.139	0.173	−0.803	0.422	
H1d	Health behavior intentions → Health behaviors	0.263	0.057	4.603	***	Tenable	0.001	0.069	0.020	0.984	Untenable

H1 is partially founded, and H2 is tenable.

** Significant at 0.01 (both sides); *** Significant at 0.001 (both sides).

It can be seen from the above comparative analysis that, first, compared to the male office group, various factors had fewer negative effects on women, showing that women were more inclusive of the environment and satisfied with various building strategies. Second, 5 and 6 variables significantly affected the health behavior intentions of the two groups, while 7 and 6 variables significantly affected their health behaviors. Moreover, behavioral intentions had a significant positive effect on men’s health behaviors but had no significant effect on women’s health behaviors. Although female office groups were easily affected by the environment to produce behavioral intentions, their purposes and actions were weaker, and their actual implementation was inferior to that of men. Furthermore, the same building environment strategies had different effects on male and female groups. Design strategies for result feedback had a significant negative effect on men’s health behaviors, but significantly promoted women’s health behaviors. In all likelihood, women were easily affected by the environment and the outside world, and continuous information feedback could stimulate their willingness to regularly implement better health behaviors. The design strategies of intelligent automation could significantly promote men’s health behaviors but had no significant effect on women. Most men were devoted to researching new technologies and products and were more eager for control and flexibility, making them more curious and interested in intelligent automation products. Optimization could significantly promote female behavior, but it had a significant negative effect on male behavior. Women have higher context sensitivity (Miller and Ubeda, 2012) and can easily perceive the environment, atmosphere, and details. According to the previous analysis, men’s work intensity was greater, and the increase in working hours and decrease in leisure time increased their work pressure, causing them to concentrate on their work instead of the detailed design of office space. Subjective cognition, assistance and guidance, spatial planning and organization, and management had significant positive effects on the health behaviors of the two groups because the research objects of this study were office groups who received higher education, had a good subjective understanding of health behaviors, and could better comply with and respond to the organization and management regulations formulated by the company. A good and healthy environment atmosphere was created through assistance, guidance, and spatial planning during the work and rest. Behavior implementation can be promoted by providing convenient and comfortable venues to meet the needs of health behaviors.

According to the multiple structural equation models and previous analysis, it was assumed that H1 and H2 were partially tenable.

## 5. Discussion

### 5.1. Discussion of the results

#### 5.1.1. Gender difference characteristics of the office groups

Analysis of the questionnaire data revealed that there are differences in the individual characteristics of the male and female office groups and the factors that affect health behaviors. Regarding individual characteristics, the results show that men’s work intensity is higher and eye diseases can occur more easily. The incidence of musculoskeletal diseases in women is higher but far lower than that in men. The proportion of insomnia and obesity was higher in women than in men. This result is inconsistent with that of other studies. Weekes et al. found that professional women experience greater stress (Lottrup et al., 2013). Most likely, the two groups had different cognitions and tolerances to stress, and the data collected by the questionnaire had some deviations. It is difficult for women to completely separate their work from their homes (Gao et al., 2022). The female stress in this study is not only limited to work intensity but also includes the work pressure imposed by women and their families. In addition, Gao et al. (2022) reported that the proportion of eye and musculoskeletal pain in women was higher than that in men. Nogueira et al. (2020) also reported that the obesity rate is higher in women, consistent with the results of this study. Most likely, both studies collected data using questionnaires, and women had a higher requirement for staying in shape.

In exploring the health behavior factors that influence both male and female office workers, it was also found that, except for attitudes and its related variables, other variables are significantly correlated with gender, in that gender determines the preferences of office individuals for organization and management strategies and objective planning strategies. These findings are generally consistent with those of previous studies, and different environmental strategies and social groups have different effects on both groups (Gao et al., 2022). Moreover, female staff members have higher expectations and satisfaction requirements for healthy building environments (Wang et al., 2022) and various intervention strategies are more effective for female staff. For example, depression in men is less affected by environmental factors than in women (Koohsari et al., 2019), and women’s sports activities and walking behaviors are usually more sensitive to the building environment (Basu et al., 2021; Yu et al., 2021). Although the supportive environment and social capital that can promote sports activities have positive and negative interaction effects on individual sports activities, the positive interaction effect in women is more significant than that in men (Jun and Park, 2019), and the comfort and accessibility of walking environments are more likely to reduce obesity in women (Gao et al., 2022).

#### 5.1.2. Gender differences in the effectiveness of strategies

On the one hand, this study found that organizational and management strategies had significant positive effects on the health behaviors of the two office groups, consistent with most of the studies. Koohsari et al. found in their study of depression that no built environment attributes were related to depression in men. However, in the Asian environment, men’s mental health might be more related to the social environment, such as joining clubs or participating in social activities (Koohsari et al., 2019). Lanjing et al. found that the external factors affecting the bike riding activities of the two groups included family characteristics and social environment. The more family members around you who rode bikes, the more likely you are to use them yourself (Wang et al., 2022). In particular, a few studies differed from this study. Gargiulo et al. found that well maintained and managed walkways could relieve depression in women but had no effect on men (Wang et al., 2022). Zheng et al. (2020) demonstrated that men’s health modes were more easily affected by the objective environment, whereas women were more easily affected by social activities (Zheng et al., 2020).

On the other hand, for design strategies in general, objective design strategies were more effective in promoting the health behaviors of the male office group (only one objective strategy had a negative effect), while subjective design strategies had the greatest effect on the health behaviors of the female office group (three subjective strategies had a significant positive effect). Separately, the effectiveness of subjective design strategies is as follows: there was a significant, positive correlation between assistance and guidance strategies and the health behaviors of the two office groups, while the effects of result feedback strategies were the opposite. Based on significantly promoting the health behaviors of female staff, it had a significant negative effect on the health behaviors of male staff. Currently, the conclusion about assistance and guidance strategies is consistent with the report of Tcymbal et al. that an environment with perfect infrastructure and user-friendly activities can promote the physical activities of both male and female staff (Tcymbal et al., 2020). However, other studies have found that the design strategies of assistance and guidance affect female behavior to a greater degree. Mitra, De Bacquer, Adlakha, Gargiulo, et al. found that excellent bicycle facilities, an environment with good lighting and safe roads could cause more female office groups to choose to ride bicycles, commute on foot and engage in more sports activities (De Cocker et al., 2015; Mitra and Nash, 2019; Adlakha and Parra, 2020; Gargiulo et al., 2020). Koohsari et al. (2019) found that a walking environment with good connectivity and perfect entertainment facilities could reduce the incidence of depression among women. Ma et al. observed a different phenomenon. Compared with men, strategies of notification and publicity, such as promoting and publicizing bicycle activities, were more helpful in improving women’s mental health and life satisfaction (Koohsari et al., 2019).

Among objective design strategies, the conclusions are as follows: spatial planning could significantly promote the health behaviors of male and female staff, whereas intelligent automation and detail optimization only promoted healthy behaviors among male and female staff, respectively. Detailed optimization had a significant negative effect on the health behaviors of the male office group. In a study of spatial planning, similar to the results of this study, Kim et al. reported that a reasonable office space layout and a combination of private and open office spaces were beneficial to the health of the two groups (Yu et al., 2021). Lottrup et al. (2013) found that planning and designing garden spaces and windows in the workplace could relieve stress in male staff. Wende et al. (2012) found that the number and planning of fast-food restaurants significantly affected obesity and dietary behaviors in men. When analyzing the effectiveness of detailed optimization strategies, Nawrath and Yu Jiabin found that increasing natural and esthetic elements and improving green street landscapes had more significant effects on promoting women’s riding and sports activities (Nawrath et al., 2019; Yu et al., 2021). Lee et al. (2018) found that ergonomic and personalized office environment designs could improve the health and performance of female staff. Contrary to the conclusions of this study, Lottrup et al. (2013) found that detailed optimization strategies, such as increasing green plants in an office, could only significantly relieve the stress of male staff but had no significant relationship with reducing stress in women. Jiang et al. (2014) also reported that different densities of tree coverage had nothing to do with women’s stress relief but could increase men’s stress relief. Most likely, their studies focused on staff stress rather than related health behaviors. For example, green plants can not only relieve stress but also promote sports activities (Nawrath et al., 2019).

### 5.2. Implications

This research has several advantages and implications. First, it focuses on testing the actual effectiveness of architectural strategies in the evaluation standard of healthy buildings on human health behaviors and increases attention to the validity of strategies themselves and behavioral orientation. In the study and collation of several healthy building evaluation standards, it was found that the internal strategies of the evaluation systems are mostly proposed from the perspective of medical principles and health guidelines, focusing on the impact of buildings on people, but lack of expression of individual behaviors affecting people’s health. Therefore, from the perspective of users, this study proposes an optimization direction for healthy building evaluation standards and rating systems, which will help designers, engineers, and operators create a healthier space environment. Second, this study complements and refines the strategies for different subjects and groups that are briefly mentioned in relevant standards. A healthy building certification includes the assessment of residential buildings, office buildings, hospitals, and other public building spaces. Different building spaces have different users, and their group characteristics, daily activities, and health needs differ. Therefore, based on the existing rating system, this study focuses on the impact of strategies on the health behaviors of office groups and examines gender differences. In addition, it complements the research methods in the field of architectural design in an interdisciplinary manner; that is, the theoretical model of planned behavior in the field of social psychology is applied to the field of architecture, and it is found that the model is applicable and the path is valid. Finally, this study adds a new case for the promotion of healthy buildings.

### 5.3. Limitations and future directions

However, this study has its shortcomings and limitations. First, only the gender difference grouping model was considered and reviewed. Although the study results proved that there were gender differences in the health behavior models for the office groups, the differences among office groups of different ages, educational backgrounds, and incomes were mixed, and these individual factors also influenced the specific implementation of health behaviors to a greater or lesser extent. At the same time, while the samples in this study include the two gender roles most commonly found in Chinese society today, further research in different settings may need to include more comprehensive gender options. In addition, the research area of this study was the Nanshan District of Shenzhen City, and a broader sample of research objects was not considered. Therefore, future studies can further explore the role of other individuals’ socioeconomic characteristics and more comprehensive gender roles in the formation mechanisms of health behaviors. Meanwhile, it is necessary to conduct a comparative analysis of two or more groups in the study area, emphasize the universality of the study, and improve its objectivity and credibility. More importantly, future studies should consider optimizing the theoretical model of planned behavior used in this study, supplementing other relevant variables, standardizing and unifying the standards of diseases and bad habits in the questionnaire, and enhancing the scientific nature of the research methods and data.

## 6. Conclusion

Based on TPB theory, this study incorporates gender differences into the mechanism for the effects of the built environment on health behaviors and establishes and compares structural equation models for both male and female office groups. It was found that the reliability and validity of the models are basically sound, the data reliability was high, and most of the model paths and research assumptions are tenable. In the direction of health behavior genesis, assumptions H1a and H1c in the first-order variable model are true, and assumptions H1b and H1d are true in women and men, respectively. H1a, H1b, and H1 in the second-variable model are partially true in both populations, assuming that H1d is true only in men. In the direction of gender difference comparison, it is assumed that H2 is true; that is, gender has a certain influence on the variables and path of health behavior formation. Therefore, it is feasible to apply TPB to building design and a healthy building evaluation system for the first time in this study. This study deeply explored and examined gender differences, not only by supplementing the basic characteristics of male and female office groups but also by finding that gender plays an important role in the office environment and health behaviors. It can also effectively predict the psychological and gender factors that affect the implementation of health behaviors by office groups and evaluate the actual effectiveness of healthy building strategies in promoting the health behaviors of different groups. This study will help optimize the WELL scoring system and related building evaluation standards, promote, and implement the development and differentiated design of healthy office buildings, and more comprehensively enhance the health levels of office groups.

Our findings show that organization and management strategies, subjective design strategies for assistance and guidance, and objective design strategies for spatial planning can significantly promote the health behaviors of the two groups. Therefore, operators, designers, and builders are encouraged to promote and implement these three strategies. In addition, the result feedback strategies only have a significant, positive effect on female behaviors, so we can also consider information feedback modes from the perspective of women, such as enhancing the esthetics of e-mail and web page layouts. However, intelligent automation can only significantly promote the health behaviors of male office workers. Automation design, such as ventilation and disinfection, can be conducted in office environments, and intelligent trash cans can be added near male staff stations to implement healthy behaviors. The detailed optimization design strategies had opposite effects on the two groups. Therefore, ergonomic fitness stations and flexible offices should be provided according to gender and individual needs to promote physical exercise by staff. Flexible plate sizes should be used to promote a healthy diet. Green plants and other nature-friendly designs can be added near women’s desks to help relieve stress.

To optimize the evaluation system of WELL healthy buildings, designers, developers, and builders can actively adopt office design strategies that promote health behaviors by adjusting the score proportions of the strategy regulations. For example, the scores of spatial planning and design strategies and organization and management strategies can be greatly improved, and the evaluation scores of the design strategies of notification and publicity can be appropriately reduced. Moreover, different strategies had different effects on the two office groups, indicating the need for differences in demand management and design. For the evaluation standards of healthy buildings, such as WELL, which only starts from a wide range of scientific principles and pathology but does not consider the needs of different groups, we can appropriately provide an additional bonus point mechanism for specific groups and gender differences, encourage more user groups to implement health behaviors and improve the actual effectiveness of different strategies.

Based on this study, we found that the current research on healthy building strategies and user behaviors needs to be expanded, and there are few studies on the interaction of health behaviors, special groups, and gender differences in user systems. As COVID-19 negatively impacts people’s health in many ways, companies and organizations are grappling with the challenge of attracting employees back to their offices. Therefore, this study calls for future design and research on the built environment to shift attention from the level of individual health behaviors to more systematic health behaviors, and it is necessary to consider the preferences and habits of special users, as well as the universality of gender-differentiated management and design. In addition, this study also suggests that future research should optimize the model architecture of TPB theory and its applicability in the field of architecture, supplement the influence variables, and further explore the potential determinants of complex design, implementation, and evaluation strategies related to health behaviors that influence multiple interrelated health outcomes.

## Data availability statement

The original contributions presented in the study are included in the article/Supplementary material, further inquiries can be directed to the corresponding author.

## Ethics statement

The requirement of ethical approval was waived by Ethics Review Committee of Shenzhen University for the studies involving humans because According to Article 32 of Chapter III of the Measures for the Ethical Review of Life Science and Medical Research Involving Human Beings issued by the China Science and Technology Ethics Committee, scientific research with the following conditions can be exempted from ethical review to reduce the unnecessary burden on researchers: (1) Conduct research using anonymized information data; (2) Does not cause harm to the human body; (3) Do not involve sensitive personal information or commercial interests. The studies were conducted in accordance with the local legislation and institutional requirements. The ethics committee/institutional review board also waived the requirement of written informed consent for participation from the participants or the participants’ legal guardians/next of kin because we clearly inform participants in the questionnaire that they have the right to participate voluntarily and can withdraw at any time without any negative consequences. We respect the autonomy of participants and ensure that their participation is based on voluntary principles. Respondents can fill in the questionnaire only after they check the “agree” option in the questionnaire.

## Author contributions

XX: conceptualization, data curation, formal analysis, methodology, resources, software, and writing—original draft. RW: methodology, investigation, data curation, visualization, software, and writing—original draft. ZG: conceptualization, methodology, validation, supervision, and resources. SC: data analysis, resources, supervision, and resources. XX, RW, ZG, and SC: writing—review and editing. All authors contributed to the article and approved the submitted version.
