# Supplementary material for: A comparative study of gender differences in healthy office building strategies
Source: Front Psychol. 2023 Nov 27;14:1146260. doi: 10.3389/fpsyg.2023.1146260 (PMC10712562; doi:10.3389/fpsyg.2023.1146260)
Supplement: Supplementary file 1 [file Table_1.DOCX]

Supplementary Material

Article Title

Xiaohuan Xie*, Ruobing Wang, Zhonghua Gou, Shan Chen*

*** Correspondence:** Shan Chen: [c_shan@szu.edu.cn](mailto:c_shan@szu.edu.cn)

# Supplementary Tables

**Supplementary Table 1.** Basic Components of User Questionnaire Scale

| **Main Aspects** | **Main Variables** | **Question Content** | |
| --- | --- | --- | --- |
| **Basic information** | Gender, age, educational background, work intensity, physical condition, poor lifestyle habits | | |
| **Attitudes** | Cognitive level | A1: Is it important/beneficial for you to implement health behaviors? | |
|  |  | A2: Does implementing health behaviors make you feel good? | |
|  | Subjective design strategies | Informative publicity | B1: Designing visible no-smoking signs and signs that describe the dangers of smoking can enhance your desire to quit smoking. |
|  |  |  | B2: Reminding employees of their sedentary duration on computer monitors can encourage you to be less sedentary and more active. |
|  |  |  | B3: Designing slogans and posters to encourage stair use can enhance your desire to climb stairs. |
|  |  |  | B4: Designing educational posters, brochures, and health menus to promote nutritious diets can enhance your desire to eat healthy. |
|  |  | Assisted guidance | C1: Aesthetically designed staircases with good quality stairwell environments can encourage you to climb more stairs. |
|  |  |  | C2: The provision of changing rooms, showers, and free fitness facilities can encourage you to work out more. |
|  |  |  | C3: Convenient access environments and beautiful exercise paths can encourage you to work out more. |
|  |  | Result feedback | D1: Setting up a display screen or designing a specific website to show real-time changes in air quality can enhance your desire to ventilate more often. |
|  |  |  | D2: Setting up displays near water fountains to show changes in water quality can encourage you to drink more water. |
|  |  |  | D3: Regular issuance of health digital briefings that summarize your recent energy consumption and health indicators can enhance your desire to live a healthy lifestyle. |
| **Subjective norms** | Social interpersonal relationship | E1: How do interpersonal relationships based on kinship or active associations, such as family and friends, affect your attitudes toward health behaviors? | |
|  |  | E2: How do other passive interpersonal relationships, such as classmates and colleagues, affect your attitudes toward health behaviors? | |
|  |  | E3: How do the information and messages that you receive from social networking platforms or software affect your attitudes toward health behaviors? | |
|  | Organizational management strategy | J1: The company’s requirements that employees should rotate their workstations every day and can choose their own work areas can encourage you to walk more and sit less. | |
|  |  | J2: Prohibiting food from being brought to workstations can encourage you to go out on foot or choose the dining area for meals, making you walk more. | |
|  |  | J3: The company’s provision of mental health services (such as electronic programs, professional psychological counselors) and regular psychological counseling for employees can help you to relieve stress. | |
|  |  | J4: The company’s provision of nutritional counselors can encourage you to have nutritious meals and healthy diets. | |
|  |  | J5: The company’s encouraging of weight management and working out, requiring employees to participate in exercise programs, such as online applets and step log feedback, and providing rewards can encourage you to walk and exercise more. | |
| **Perceived behavioral control** | Self-efficacy perception | H1: How easy or difficult is it for you to implement health behaviors (with the necessary resources, time, and opportunities)? | |
|  |  | H2: How easy is it for you to implement health behaviors (with the necessary resources, time, opportunities, etc.)? | |
|  | Objective design strategies | Spatial planning | I1: Placing water fountains in commonly used floor spaces and in each dining area encourages you to drink more water. |
|  |  |  | I2: Providing and optimizing space for sidewalks, bicycle paths, and bicycle parking can encourage your green travel, workout and fitness. |
|  |  |  | I3: Designing stairs in highly visible spaces (such as building entrances and atriums) and interconnected stairs on working floors to facilitate short meetings with colleagues across floors can encourage you to climb stairs. |
|  |  |  | I4: Placing fruits and vegetables in prominent places for sale or eating can encourage you to eat more fruits and vegetables. |
|  |  | Detail optimization | G1: Providing and designing windows that can be opened to connect the indoors with the outdoor environment can encourage your regular ventilation. |
|  |  |  | G2: Designing food labels with information about nutrition, ingredients, and optimizing the size of tableware to guide proper portion sizes can encourage your healthy diet. |
|  |  |  | G3: Designing desks and chairs based on ergonomics and fitness workstations can encourage you to work in a standing position. |
|  |  |  | G4: Integrating aesthetics and design and adding artistic and pleasing elements can help you to manage and relieve stress. |
|  |  | Intelligent automation | K1: Designing mechanical ventilation systems can encourage your regular ventilation. |
|  |  |  | K2: Designing air conditioners with low emissions and energy consumption, water saving devices, energy-saving intelligent lights, etc., can encourage you to choose to buy similar green products for your family. |
|  |  |  | K3: Intelligent design of garbage cans (which can identify the type of garbage, open and close the lid automatically, etc.) can encourage your garbage sorting behavior. |
| **Health behavior intentions** | L1: Are you willing to implement health behaviors? | | |
|  | L2: Do you plan to start working on health behaviors soon? | | |
|  | L3: Will you encourage others to practice health behaviors in the future? | | |
|  | L4: Do you plan to learn more about health behaviors in the future through social media (such as TV, internet, and newspapers)? | | |
|  | L5: Do you plan to use social media to help you to implement health behaviors in the future (such as following exercise bloggers to work out regularly, keeping a daily diet according to online nutrition menus)? | | |
| **Health behaviors** | M1: Did you exercise and work out regularly? | | |
|  | M2: Did you ventilate your office regularly? | | |
|  | M3: Did you drink water often? | | |
|  | M4: Did you always keep a healthy diet and pay attention to balanced nutrition? | | |
|  | M5：Did you often perform stress management and relief activities? | | |
|  | M6: Did you often use natural lighting? | | |
